# 1-Methyl-2-({[(1-methyl-1*H*-benzimid­azol-2-yl)meth­yl](phen­yl)amino}­meth­yl)1*H*-benzimidazol-3-ium picrate

**DOI:** 10.1107/S1600536811024275

**Published:** 2011-06-25

**Authors:** Bin Liu, Fan Kou, Fei Jia, Jingkui Yuan, Huilu Wu

**Affiliations:** a School of Chemical and Biological Engineering, Lanzhou Jiaotong University, Lanzhou 730070, People’s Republic of China

## Abstract

In the title molecular salt, C_24_H_24_N_5_^+^·C_6_H_2_N_3_O_7_^−^, the dihedral angle between the benzimidazole rings of the cation is 5.041 (2)°. In the anion, the three nitro groups make dihedral angles of 2.468 (3), 12.795 (3) and 24.958 (4)° with respect to the central ring. In the crystal, weak aromatic π–π stacking [centroid–centroid distance = 3.599 (15) Å] consolidates the packing. In addition, an intra­molecular N—H⋯N hydrogen bond is observed.

## Related literature

For background to proton-transfer compounds, see: Aghabozorg *et al.* (2008 ▶) and to benzimidazoles, see: Ram *et al.* (1992 ▶). For the biological activivity of benzimidazoles, see: Baraldi *et al.* (2004 ▶); Göker *et al.* (2002 ▶); Jayasekera *et al.* (2005 ▶); Starčević *et al.* (2007 ▶). 
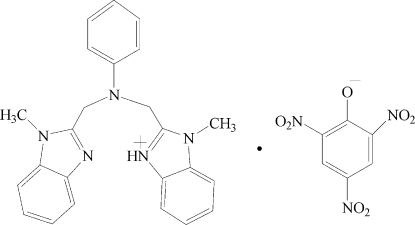


## Experimental

### 

#### Crystal data

C_24_H_24_N_5_^+^·C_6_H_2_N_3_O_7_^−^
*M* *_r_* = 610.59
Triclinic, 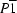

*a* = 9.4233 (5) Å
*b* = 12.3523 (7) Å
*c* = 12.5772 (7) Å
α = 92.007 (1)°
β = 98.497 (1)°
γ = 103.685 (1)°
*V* = 1403.07 (13) Å^3^
*Z* = 2
Mo *K*α radiation
μ = 0.11 mm^−1^
*T* = 293 K
0.31 × 0.30 × 0.29 mm

#### Data collection

Bruker SMART APEX diffractometer
Absorption correction: multi-scan (*SADABS*; Sheldrick, 2001 ▶) *T*_min_ = 0.968, *T*_max_ = 0.970
11689 measured reflections
5217 independent reflections
3567 reflections with *I* > 2σ(*I*)
*R*_int_ = 0.025

#### Refinement

*R*[*F* ^2^ > 2σ(*F* ^2^)] = 0.062
*wR*(*F* ^2^) = 0.221
*S* = 1.19
5217 reflections
411 parameters
1 restraint
H atoms treated by a mixture of independent and constrained refinement
Δρ_max_ = 0.60 e Å^−3^
Δρ_min_ = −0.50 e Å^−3^

### 

Data collection: *SMART* (Bruker, 2001 ▶); cell refinement: *SAINT* (Bruker, 2001 ▶); data reduction: *SAINT*; program(s) used to solve structure: *SHELXS97* (Sheldrick, 2008 ▶); program(s) used to refine structure: *SHELXL97* (Sheldrick, 2008 ▶); molecular graphics: *SHELXTL* (Sheldrick, 2008 ▶); software used to prepare material for publication: *SHELXTL*.

## Supplementary Material

Crystal structure: contains datablock(s) global, I. DOI: 10.1107/S1600536811024275/lr2014sup1.cif

Structure factors: contains datablock(s) I. DOI: 10.1107/S1600536811024275/lr2014Isup2.hkl

Supplementary material file. DOI: 10.1107/S1600536811024275/lr2014Isup3.cml

Additional supplementary materials:  crystallographic information; 3D view; checkCIF report

## Figures and Tables

**Table 1:** Hydrogen-bond geometry (Å, °)

*D*—H⋯*A*	*D*—H	H⋯*A*	*D*⋯*A*	*D*—H⋯*A*
N3—H3*N*⋯N1	0.92	1.85	2.715 (8)	157

**Fig. 1.**
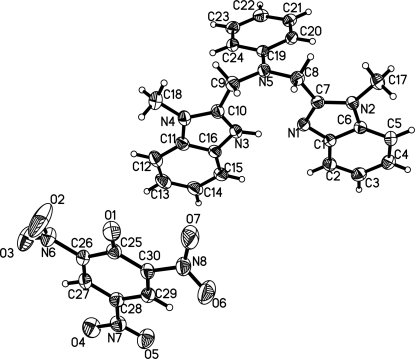
The molecular structure of the title compound.Displacement ellipsoids are drawn at the 30% probability level.

**Fig. 2.**
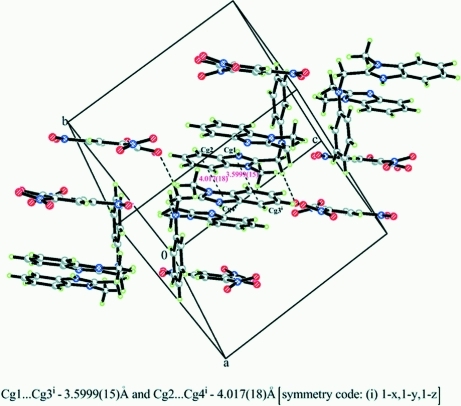
A view of the crystal packing of the title compound. Intermolecular hydrogen bonds are shown as dashed lines.

## supplementary crystallographic information

### Comment

Bis-benzimidazoles are known to be strong chelating agents coordinating through both the C=N nitrogen atoms. In addition, bis-benzimidazoles have a polymer-forming characteristic as a typical multidentate ligand. Benzimidazole compounds are environmentally friendly compounds with two high active nitrogen atoms in 1, 3-sites (Ram *et al.*, 1992). Benzimidazoles and their azino-fused cyclic derivatives have a wide range of well known biological activities such as anticancer (Baraldi *et al.*, 2004), antimicrobial (Jayasekera *et al.*, 2005), antifungal (Göker *et al.*, 2002), antiviral (Starčević *et al.*, 2007).

In this paper, the asymmetric unit of the title proton transfer compound consists of a bis(*N*-methylbenzimidazol-2-ylmethyl)aniline(MEBBA) cation interacting with a picrate anion. The proton of the picric acid is transfered to the N3 nitrogen atoms of the MEBBA(Fig. 1). The dihedral angle between the planes defined by N2—C7—N1 and N3—C10—N4 is 5.041 (2)°, which indicates that the two benzimidazole rings are almost coplanar.

The crystal structure is mainly stabilized by weak π–π interactions involving the benzimidazol rings with centroid-centroid distances, *Cg*1 ··· *Cg* 3^i^ and *Cg*2 ···*Cg*4^i^ of 3.5999 (15) and 4.017 (18) Å repectively [symmetry code: (i) 1-x,1-y,1-z. *Cg*1 centroid of the (N1,C1,C6,N2,C7) ring; *Cg*2 centroid of the (N3,C10,N4.C11,C16) ring; *Cg*3 centroid of the (C11-C16) ring; Cg4 centroid of the (C1-C6) ring ]. In addition an N3-H3N···N1 intramolecular hydrogen bond is observed.

### Refinement

All H atoms were geometrically positioned and refined using a riding-model approximation with C—H distances from 0.93 to 0.97 Å and N—H = 0.92 Å, and with *U*_iso_(H) = 1.2 *U*_eq_(C) or *U*_iso_(H) = 1.5 *U*_eq_(C_methyl_) or *U*_iso_(H) = 1.1 *U*_eq_(N).

### Crystal data

**Table:**

C_30_H_26_N_8_O_7_	*Z* = 2
*M**_r_* = 610.59	*F*(000) = 636
Triclinic, *P*1	*D*_x_ = 1.445 Mg m^−^^3^
*a* = 9.4233 (5) Å	Mo *K*α radiation, λ = 0.71073 Å
*b* = 12.3523 (7) Å	Cell parameters from 5217 reflections
*c* = 12.5772 (7) Å	θ = 3.0–25.5°
α = 92.007 (1)°	µ = 0.11 mm^−^^1^
β = 98.497 (1)°	*T* = 293 K
γ = 103.685 (1)°	Block, yellow
*V* = 1403.07 (13) Å^3^	0.31 × 0.30 × 0.29 mm

### Data collection

**Table:**

Bruker SMART APEX diffractometer	5217 independent reflections
Radiation source: fine-focus sealed tube	3567 reflections with *I* > 2σ(*I*)
graphite	*R*_int_ = 0.025
ω scans	θ_max_ = 25.5°, θ_min_ = 3.0°
Absorption correction: multi-scan (*SADABS*; Sheldrick, 2001)	*h* = −11→11
*T*_min_ = 0.968, *T*_max_ = 0.970	*k* = −14→14
11689 measured reflections	*l* = −15→13

### Refinement

**Table:**

Refinement on *F*^2^	Secondary atom site location: difference Fourier map
Least-squares matrix: full	Hydrogen site location: inferred from neighbouring sites
*R*[*F*^2^ > 2σ(*F*^2^)] = 0.062	H atoms treated by a mixture of independent and constrained refinement
*wR*(*F*^2^) = 0.221	*w* = 1/[σ^2^(*F*_o_^2^) + (0.1022*P*)^2^ + 0.6611*P*] where *P* = (*F*_o_^2^ + 2*F*_c_^2^)/3
*S* = 1.19	(Δ/σ)_max_ < 0.001
5217 reflections	Δρ_max_ = 0.60 e Å^−^^3^
411 parameters	Δρ_min_ = −0.50 e Å^−^^3^
1 restraint	Extinction correction: *SHELXL97* (Sheldrick, 2008), Fc^*^=kFc[1+0.001xFc^2^λ^3^/sin(2θ)]^-1/4^
Primary atom site location: structure-invariant direct methods	Extinction coefficient: 0.024 (4)

### Special details

**Table:**

Geometry. All e.s.d.'s (except the e.s.d. in the dihedral angle between two l.s. planes) are estimated using the full covariance matrix. The cell e.s.d.'s are taken into account individually in the estimation of e.s.d.'s in distances, angles and torsion angles; correlations between e.s.d.'s in cell parameters are only used when they are defined by crystal symmetry. An approximate (isotropic) treatment of cell e.s.d.'s is used for estimating e.s.d.'s involving l.s. planes.
Refinement. Refinement of *F*^2^ against ALL reflections. The weighted *R*-factor *wR* and goodness of fit *S* are based on *F*^2^, conventional *R*-factors *R* are based on *F*, with *F* set to zero for negative *F*^2^. The threshold expression of *F*^2^ > σ(*F*^2^) is used only for calculating *R*-factors(gt) *etc*. and is not relevant to the choice of reflections for refinement. *R*-factors based on *F*^2^ are statistically about twice as large as those based on *F*, and *R*- factors based on ALL data will be even larger.

### Fractional atomic coordinates and isotropic or equivalent isotropic displacement parameters (Å^2^)

**Table:**

	*x*	*y*	*z*	*U*_iso_*/*U*_eq_	
O1	0.4994 (3)	−0.13472 (19)	0.29886 (19)	0.0706 (7)	
O2	0.2431 (4)	−0.2821 (4)	0.2686 (4)	0.172 (2)	
O3	0.0611 (3)	−0.2466 (3)	0.1765 (3)	0.1027 (10)	
O4	0.1139 (3)	0.0278 (2)	−0.0786 (2)	0.0864 (8)	
O5	0.3145 (3)	0.1577 (2)	−0.0626 (2)	0.0874 (9)	
O6	0.7374 (3)	0.1381 (3)	0.1867 (3)	0.1111 (12)	
O7	0.7036 (3)	0.0635 (3)	0.3324 (3)	0.1153 (13)	
N1	0.5570 (3)	0.6424 (2)	0.67349 (18)	0.0486 (6)	
N2	0.7671 (2)	0.7189 (2)	0.78335 (18)	0.0474 (6)	
N3	0.3314 (3)	0.4630 (2)	0.59716 (19)	0.0518 (6)	
N4	0.2023 (3)	0.2910 (2)	0.6013 (2)	0.0530 (6)	
N5	0.4792 (3)	0.46665 (19)	0.81228 (19)	0.0481 (6)	
N6	0.1909 (3)	−0.2216 (2)	0.2111 (3)	0.0674 (8)	
N7	0.2401 (3)	0.0720 (2)	−0.0337 (2)	0.0610 (7)	
N8	0.6589 (3)	0.0772 (2)	0.2407 (2)	0.0643 (7)	
C1	0.6137 (3)	0.7487 (2)	0.6418 (2)	0.0465 (7)	
C2	0.5602 (4)	0.8062 (3)	0.5586 (2)	0.0570 (8)	
H2A	0.4724	0.7746	0.5123	0.068*	
C3	0.6420 (4)	0.9119 (3)	0.5471 (3)	0.0650 (9)	
H3A	0.6086	0.9527	0.4922	0.078*	
C4	0.7734 (4)	0.9591 (3)	0.6158 (3)	0.0658 (9)	
H4A	0.8254	1.0309	0.6056	0.079*	
C5	0.8294 (4)	0.9035 (3)	0.6984 (3)	0.0593 (8)	
H5A	0.9181	0.9353	0.7436	0.071*	
C6	0.7461 (3)	0.7972 (2)	0.7104 (2)	0.0476 (7)	
C7	0.6516 (3)	0.6286 (2)	0.7579 (2)	0.0467 (7)	
C8	0.6330 (3)	0.5244 (3)	0.8170 (2)	0.0506 (7)	
H8A	0.6844	0.4747	0.7862	0.061*	
H8B	0.6782	0.5435	0.8918	0.061*	
C9	0.4206 (3)	0.3610 (2)	0.7497 (2)	0.0542 (7)	
H9A	0.3671	0.3075	0.7934	0.065*	
H9B	0.5019	0.3328	0.7306	0.065*	
C10	0.3194 (3)	0.3709 (2)	0.6493 (2)	0.0496 (7)	
C11	0.1347 (3)	0.3364 (3)	0.5125 (2)	0.0532 (7)	
C12	0.0087 (4)	0.2915 (3)	0.4368 (3)	0.0662 (9)	
H12A	−0.0465	0.2185	0.4375	0.079*	
C13	−0.0292 (4)	0.3618 (4)	0.3608 (3)	0.0736 (11)	
H13A	−0.1131	0.3356	0.3090	0.088*	
C14	0.0544 (4)	0.4711 (3)	0.3591 (3)	0.0696 (9)	
H14A	0.0251	0.5157	0.3062	0.084*	
C15	0.1784 (4)	0.5142 (3)	0.4334 (2)	0.0593 (8)	
H15A	0.2341	0.5870	0.4321	0.071*	
C16	0.2174 (3)	0.4447 (3)	0.5106 (2)	0.0506 (7)	
C17	0.8923 (3)	0.7329 (3)	0.8698 (3)	0.0642 (9)	
H17A	0.8817	0.6669	0.9089	0.096*	
H17B	0.8956	0.7960	0.9178	0.096*	
H17C	0.9823	0.7452	0.8399	0.096*	
C18	0.1542 (4)	0.1791 (3)	0.6371 (3)	0.0715 (10)	
H18A	0.2203	0.1705	0.7003	0.107*	
H18B	0.1548	0.1247	0.5809	0.107*	
H18C	0.0558	0.1685	0.6537	0.107*	
C19	0.3877 (3)	0.5143 (2)	0.8665 (2)	0.0434 (6)	
C20	0.4428 (3)	0.6143 (2)	0.9302 (2)	0.0495 (7)	
H20A	0.5427	0.6500	0.9368	0.059*	
C21	0.3521 (4)	0.6609 (3)	0.9831 (2)	0.0564 (8)	
H21A	0.3918	0.7272	1.0260	0.068*	
C22	0.2039 (4)	0.6114 (3)	0.9739 (3)	0.0590 (8)	
H22A	0.1425	0.6441	1.0088	0.071*	
C23	0.1481 (3)	0.5121 (3)	0.9119 (2)	0.0601 (8)	
H23A	0.0478	0.4776	0.9052	0.072*	
C24	0.2373 (3)	0.4627 (3)	0.8597 (2)	0.0530 (7)	
H24A	0.1975	0.3947	0.8197	0.064*	
C25	0.4378 (3)	−0.0849 (2)	0.2298 (2)	0.0489 (7)	
C26	0.2853 (3)	−0.1251 (2)	0.1753 (2)	0.0482 (7)	
C27	0.2223 (3)	−0.0748 (2)	0.0922 (2)	0.0504 (7)	
H27A	0.1246	−0.1048	0.0603	0.060*	
C28	0.3037 (3)	0.0202 (2)	0.0560 (2)	0.0498 (7)	
C29	0.4476 (3)	0.0692 (2)	0.1063 (2)	0.0515 (7)	
H29A	0.5011	0.1347	0.0827	0.062*	
C30	0.5096 (3)	0.0199 (2)	0.1907 (2)	0.0483 (7)	
H3N	0.398 (2)	0.5313 (13)	0.606 (2)	0.046 (8)*	

### Atomic displacement parameters (Å^2^)

**Table:**

	*U*^11^	*U*^22^	*U*^33^	*U*^12^	*U*^13^	*U*^23^
O1	0.0762 (15)	0.0589 (14)	0.0696 (15)	0.0146 (12)	−0.0099 (12)	0.0146 (11)
O2	0.103 (3)	0.148 (4)	0.237 (5)	−0.016 (2)	−0.012 (3)	0.137 (4)
O3	0.0588 (17)	0.091 (2)	0.148 (3)	−0.0060 (15)	0.0184 (17)	0.032 (2)
O4	0.0647 (16)	0.102 (2)	0.0810 (18)	0.0148 (15)	−0.0184 (13)	0.0133 (15)
O5	0.0880 (19)	0.0789 (18)	0.0841 (18)	0.0042 (15)	−0.0026 (15)	0.0349 (15)
O6	0.0684 (18)	0.127 (3)	0.109 (2)	−0.0245 (18)	−0.0051 (16)	0.034 (2)
O7	0.089 (2)	0.126 (3)	0.090 (2)	−0.0287 (18)	−0.0357 (17)	0.0316 (19)
N1	0.0416 (12)	0.0545 (14)	0.0457 (13)	0.0069 (10)	0.0007 (10)	0.0083 (11)
N2	0.0387 (12)	0.0566 (14)	0.0438 (12)	0.0090 (11)	0.0010 (10)	0.0047 (11)
N3	0.0533 (14)	0.0481 (14)	0.0470 (13)	0.0038 (12)	0.0006 (11)	0.0011 (11)
N4	0.0505 (14)	0.0464 (14)	0.0586 (15)	0.0070 (11)	0.0069 (11)	−0.0036 (11)
N5	0.0462 (13)	0.0451 (13)	0.0483 (13)	0.0065 (10)	0.0004 (10)	0.0049 (10)
N6	0.0639 (19)	0.0519 (16)	0.083 (2)	0.0054 (14)	0.0122 (15)	0.0138 (14)
N7	0.0594 (17)	0.0658 (18)	0.0556 (15)	0.0177 (14)	−0.0016 (13)	0.0062 (13)
N8	0.0553 (16)	0.0602 (17)	0.0666 (18)	0.0007 (13)	−0.0038 (14)	0.0076 (14)
C1	0.0437 (15)	0.0498 (16)	0.0436 (14)	0.0078 (12)	0.0049 (12)	0.0041 (12)
C2	0.0579 (18)	0.0584 (19)	0.0509 (17)	0.0108 (15)	0.0004 (14)	0.0092 (14)
C3	0.076 (2)	0.062 (2)	0.0574 (19)	0.0149 (17)	0.0125 (16)	0.0160 (16)
C4	0.074 (2)	0.0530 (19)	0.065 (2)	0.0019 (16)	0.0152 (17)	0.0096 (16)
C5	0.0535 (18)	0.0581 (19)	0.0586 (18)	0.0002 (15)	0.0077 (14)	−0.0010 (15)
C6	0.0457 (15)	0.0509 (16)	0.0445 (15)	0.0085 (13)	0.0080 (12)	0.0009 (12)
C7	0.0410 (14)	0.0563 (17)	0.0422 (14)	0.0107 (13)	0.0059 (12)	0.0055 (12)
C8	0.0403 (15)	0.0588 (18)	0.0517 (16)	0.0119 (13)	0.0034 (12)	0.0088 (13)
C9	0.0579 (18)	0.0468 (16)	0.0562 (17)	0.0133 (14)	0.0033 (14)	0.0039 (13)
C10	0.0512 (16)	0.0458 (16)	0.0497 (16)	0.0097 (13)	0.0051 (13)	0.0010 (13)
C11	0.0478 (16)	0.0621 (19)	0.0482 (16)	0.0119 (14)	0.0077 (13)	−0.0079 (14)
C12	0.0513 (18)	0.072 (2)	0.068 (2)	0.0075 (16)	0.0015 (16)	−0.0176 (18)
C13	0.0528 (19)	0.104 (3)	0.056 (2)	0.016 (2)	−0.0061 (15)	−0.016 (2)
C14	0.063 (2)	0.091 (3)	0.0509 (18)	0.0170 (19)	0.0001 (15)	−0.0035 (17)
C15	0.0578 (18)	0.069 (2)	0.0472 (16)	0.0122 (16)	0.0023 (14)	0.0031 (15)
C16	0.0464 (15)	0.0592 (18)	0.0426 (15)	0.0085 (14)	0.0041 (12)	−0.0008 (13)
C17	0.0467 (17)	0.077 (2)	0.0596 (19)	0.0074 (16)	−0.0080 (14)	0.0044 (16)
C18	0.065 (2)	0.054 (2)	0.088 (3)	−0.0007 (16)	0.0122 (18)	0.0033 (18)
C19	0.0437 (14)	0.0461 (15)	0.0377 (13)	0.0076 (12)	0.0016 (11)	0.0093 (11)
C20	0.0467 (15)	0.0487 (16)	0.0499 (16)	0.0047 (13)	0.0078 (13)	0.0104 (13)
C21	0.066 (2)	0.0519 (17)	0.0505 (17)	0.0102 (15)	0.0121 (14)	0.0113 (14)
C22	0.0565 (18)	0.074 (2)	0.0513 (17)	0.0221 (16)	0.0133 (14)	0.0115 (16)
C23	0.0446 (16)	0.081 (2)	0.0498 (17)	0.0056 (16)	0.0070 (13)	0.0132 (16)
C24	0.0460 (16)	0.0573 (18)	0.0478 (16)	0.0000 (14)	0.0015 (13)	0.0084 (13)
C25	0.0546 (17)	0.0440 (15)	0.0469 (15)	0.0132 (13)	0.0029 (13)	0.0018 (12)
C26	0.0486 (16)	0.0398 (15)	0.0540 (16)	0.0061 (12)	0.0086 (13)	0.0040 (12)
C27	0.0421 (15)	0.0465 (16)	0.0571 (17)	0.0052 (12)	0.0022 (13)	−0.0050 (13)
C28	0.0486 (16)	0.0528 (17)	0.0463 (15)	0.0130 (13)	0.0006 (12)	0.0049 (13)
C29	0.0535 (17)	0.0479 (16)	0.0508 (16)	0.0087 (13)	0.0069 (13)	0.0049 (13)
C30	0.0422 (15)	0.0466 (16)	0.0519 (16)	0.0073 (12)	0.0008 (12)	0.0022 (13)

### Geometric parameters (Å, °)

**Table:**

O1—C25	1.236 (3)	C9—H9A	0.9700
O2—N6	1.196 (4)	C9—H9B	0.9700
O3—N6	1.198 (4)	C11—C16	1.383 (4)
O4—N7	1.227 (3)	C11—C12	1.393 (4)
O5—N7	1.222 (4)	C12—C13	1.378 (5)
O6—N8	1.223 (4)	C12—H12A	0.9300
O7—N8	1.199 (4)	C13—C14	1.395 (5)
N1—C7	1.326 (3)	C13—H13A	0.9300
N1—C1	1.391 (4)	C14—C15	1.368 (4)
N2—C7	1.353 (4)	C14—H14A	0.9300
N2—C6	1.384 (4)	C15—C16	1.385 (4)
N2—C17	1.455 (4)	C15—H15A	0.9300
N3—C10	1.322 (4)	C17—H17A	0.9600
N3—C16	1.383 (4)	C17—H17B	0.9600
N3—H3N	0.916 (10)	C17—H17C	0.9600
N4—C10	1.343 (4)	C18—H18A	0.9600
N4—C11	1.403 (4)	C18—H18B	0.9600
N4—C18	1.458 (4)	C18—H18C	0.9600
N5—C19	1.393 (4)	C19—C20	1.393 (4)
N5—C9	1.444 (4)	C19—C24	1.398 (4)
N5—C8	1.447 (4)	C20—C21	1.371 (4)
N6—C26	1.438 (4)	C20—H20A	0.9300
N7—C28	1.440 (4)	C21—C22	1.372 (4)
N8—C30	1.453 (4)	C21—H21A	0.9300
C1—C2	1.385 (4)	C22—C23	1.377 (5)
C1—C6	1.397 (4)	C22—H22A	0.9300
C2—C3	1.374 (5)	C23—C24	1.373 (5)
C2—H2A	0.9300	C23—H23A	0.9300
C3—C4	1.388 (5)	C24—H24A	0.9300
C3—H3A	0.9300	C25—C30	1.450 (4)
C4—C5	1.374 (5)	C25—C26	1.460 (4)
C4—H4A	0.9300	C26—C27	1.366 (4)
C5—C6	1.387 (4)	C27—C28	1.374 (4)
C5—H5A	0.9300	C27—H27A	0.9300
C7—C8	1.495 (4)	C28—C29	1.392 (4)
C8—H8A	0.9700	C29—C30	1.368 (4)
C8—H8B	0.9700	C29—H29A	0.9300
C9—C10	1.494 (4)		
			
C7—N1—C1	106.1 (2)	C13—C12—H12A	122.0
C7—N2—C6	107.3 (2)	C11—C12—H12A	122.0
C7—N2—C17	127.3 (3)	C12—C13—C14	122.0 (3)
C6—N2—C17	125.4 (3)	C12—C13—H13A	119.0
C10—N3—C16	108.6 (3)	C14—C13—H13A	119.0
C10—N3—H3N	133.7 (18)	C15—C14—C13	121.5 (3)
C16—N3—H3N	117.7 (18)	C15—C14—H14A	119.2
C10—N4—C11	107.1 (2)	C13—C14—H14A	119.2
C10—N4—C18	125.8 (3)	C14—C15—C16	117.1 (3)
C11—N4—C18	127.1 (3)	C14—C15—H15A	121.5
C19—N5—C9	120.3 (2)	C16—C15—H15A	121.5
C19—N5—C8	119.8 (2)	C11—C16—N3	107.0 (3)
C9—N5—C8	119.9 (3)	C11—C16—C15	121.5 (3)
O2—N6—O3	119.6 (3)	N3—C16—C15	131.5 (3)
O2—N6—C26	120.2 (3)	N2—C17—H17A	109.5
O3—N6—C26	120.0 (3)	N2—C17—H17B	109.5
O5—N7—O4	122.7 (3)	H17A—C17—H17B	109.5
O5—N7—C28	118.7 (3)	N2—C17—H17C	109.5
O4—N7—C28	118.6 (3)	H17A—C17—H17C	109.5
O7—N8—O6	121.3 (3)	H17B—C17—H17C	109.5
O7—N8—C30	120.3 (3)	N4—C18—H18A	109.5
O6—N8—C30	118.4 (3)	N4—C18—H18B	109.5
C2—C1—N1	130.7 (3)	H18A—C18—H18B	109.5
C2—C1—C6	120.7 (3)	N4—C18—H18C	109.5
N1—C1—C6	108.6 (2)	H18A—C18—H18C	109.5
C3—C2—C1	117.4 (3)	H18B—C18—H18C	109.5
C3—C2—H2A	121.3	N5—C19—C20	121.3 (2)
C1—C2—H2A	121.3	N5—C19—C24	121.1 (3)
C2—C3—C4	121.4 (3)	C20—C19—C24	117.6 (3)
C2—C3—H3A	119.3	C21—C20—C19	121.1 (3)
C4—C3—H3A	119.3	C21—C20—H20A	119.5
C5—C4—C3	122.4 (3)	C19—C20—H20A	119.5
C5—C4—H4A	118.8	C20—C21—C22	121.1 (3)
C3—C4—H4A	118.8	C20—C21—H21A	119.5
C4—C5—C6	116.2 (3)	C22—C21—H21A	119.5
C4—C5—H5A	121.9	C21—C22—C23	118.5 (3)
C6—C5—H5A	121.9	C21—C22—H22A	120.7
N2—C6—C5	132.0 (3)	C23—C22—H22A	120.7
N2—C6—C1	106.0 (2)	C24—C23—C22	121.5 (3)
C5—C6—C1	122.0 (3)	C24—C23—H23A	119.3
N1—C7—N2	112.1 (2)	C22—C23—H23A	119.3
N1—C7—C8	123.7 (3)	C23—C24—C19	120.3 (3)
N2—C7—C8	124.2 (2)	C23—C24—H24A	119.9
N5—C8—C7	112.6 (2)	C19—C24—H24A	119.9
N5—C8—H8A	109.1	O1—C25—C30	124.1 (3)
C7—C8—H8A	109.1	O1—C25—C26	124.5 (3)
N5—C8—H8B	109.1	C30—C25—C26	111.4 (2)
C7—C8—H8B	109.1	C27—C26—N6	116.3 (3)
H8A—C8—H8B	107.8	C27—C26—C25	124.1 (3)
N5—C9—C10	112.1 (2)	N6—C26—C25	119.6 (3)
N5—C9—H9A	109.2	C26—C27—C28	119.8 (3)
C10—C9—H9A	109.2	C26—C27—H27A	120.1
N5—C9—H9B	109.2	C28—C27—H27A	120.1
C10—C9—H9B	109.2	C27—C28—C29	120.8 (3)
H9A—C9—H9B	107.9	C27—C28—N7	120.4 (3)
N3—C10—N4	110.6 (3)	C29—C28—N7	118.8 (3)
N3—C10—C9	123.7 (3)	C30—C29—C28	119.3 (3)
N4—C10—C9	125.7 (3)	C30—C29—H29A	120.4
C16—C11—C12	121.9 (3)	C28—C29—H29A	120.4
C16—C11—N4	106.7 (2)	C29—C30—C25	124.3 (3)
C12—C11—N4	131.4 (3)	C29—C30—N8	116.1 (3)
C13—C12—C11	116.0 (3)	C25—C30—N8	119.5 (2)
			
C7—N1—C1—C2	−179.9 (3)	N4—C11—C16—N3	−0.3 (3)
C7—N1—C1—C6	0.3 (3)	C12—C11—C16—C15	0.3 (5)
N1—C1—C2—C3	180.0 (3)	N4—C11—C16—C15	−178.7 (3)
C6—C1—C2—C3	−0.3 (5)	C10—N3—C16—C11	0.0 (3)
C1—C2—C3—C4	0.3 (5)	C10—N3—C16—C15	178.2 (3)
C2—C3—C4—C5	0.2 (6)	C14—C15—C16—C11	0.1 (5)
C3—C4—C5—C6	−0.7 (5)	C14—C15—C16—N3	−177.9 (3)
C7—N2—C6—C5	−179.6 (3)	C9—N5—C19—C20	177.9 (2)
C17—N2—C6—C5	−0.2 (5)	C8—N5—C19—C20	−3.3 (4)
C7—N2—C6—C1	0.1 (3)	C9—N5—C19—C24	−1.5 (4)
C17—N2—C6—C1	179.4 (3)	C8—N5—C19—C24	177.3 (2)
C4—C5—C6—N2	−179.6 (3)	N5—C19—C20—C21	179.8 (3)
C4—C5—C6—C1	0.8 (5)	C24—C19—C20—C21	−0.8 (4)
C2—C1—C6—N2	180.0 (3)	C19—C20—C21—C22	−0.9 (4)
N1—C1—C6—N2	−0.2 (3)	C20—C21—C22—C23	1.3 (4)
C2—C1—C6—C5	−0.3 (5)	C21—C22—C23—C24	−0.1 (5)
N1—C1—C6—C5	179.5 (3)	C22—C23—C24—C19	−1.6 (4)
C1—N1—C7—N2	−0.2 (3)	N5—C19—C24—C23	−178.6 (3)
C1—N1—C7—C8	−179.6 (3)	C20—C19—C24—C23	2.0 (4)
C6—N2—C7—N1	0.1 (3)	O2—N6—C26—C27	166.1 (4)
C17—N2—C7—N1	−179.2 (3)	O3—N6—C26—C27	−8.0 (5)
C6—N2—C7—C8	179.5 (3)	O2—N6—C26—C25	−15.6 (6)
C17—N2—C7—C8	0.1 (5)	O3—N6—C26—C25	170.3 (3)
C19—N5—C8—C7	−68.7 (3)	O1—C25—C26—C27	−173.8 (3)
C9—N5—C8—C7	110.1 (3)	C30—C25—C26—C27	4.8 (4)
N1—C7—C8—N5	−29.1 (4)	O1—C25—C26—N6	8.0 (5)
N2—C7—C8—N5	151.6 (3)	C30—C25—C26—N6	−173.4 (3)
C19—N5—C9—C10	70.9 (3)	N6—C26—C27—C28	177.7 (3)
C8—N5—C9—C10	−107.9 (3)	C25—C26—C27—C28	−0.6 (5)
C16—N3—C10—N4	0.3 (3)	C26—C27—C28—C29	−3.1 (5)
C16—N3—C10—C9	−177.8 (3)	C26—C27—C28—N7	178.0 (3)
C11—N4—C10—N3	−0.4 (3)	O5—N7—C28—C27	178.6 (3)
C18—N4—C10—N3	−179.3 (3)	O4—N7—C28—C27	−2.1 (5)
C11—N4—C10—C9	177.6 (3)	O5—N7—C28—C29	−0.3 (4)
C18—N4—C10—C9	−1.3 (5)	O4—N7—C28—C29	179.1 (3)
N5—C9—C10—N3	29.2 (4)	C27—C28—C29—C30	1.9 (5)
N5—C9—C10—N4	−148.6 (3)	N7—C28—C29—C30	−179.2 (3)
C10—N4—C11—C16	0.4 (3)	C28—C29—C30—C25	3.1 (5)
C18—N4—C11—C16	179.3 (3)	C28—C29—C30—N8	−178.6 (3)
C10—N4—C11—C12	−178.4 (3)	O1—C25—C30—C29	172.5 (3)
C18—N4—C11—C12	0.4 (5)	C26—C25—C30—C29	−6.1 (4)
C16—C11—C12—C13	−0.6 (5)	O1—C25—C30—N8	−5.7 (5)
N4—C11—C12—C13	178.1 (3)	C26—C25—C30—N8	175.7 (3)
C11—C12—C13—C14	0.6 (5)	O7—N8—C30—C29	155.6 (4)
C12—C13—C14—C15	−0.2 (6)	O6—N8—C30—C29	−25.2 (5)
C13—C14—C15—C16	−0.1 (5)	O7—N8—C30—C25	−26.1 (5)
C12—C11—C16—N3	178.7 (3)	O6—N8—C30—C25	153.1 (3)

### Hydrogen-bond geometry (Å, °)

**Table:**

*D*—H···*A*	*D*—H	H···*A*	*D*···*A*	*D*—H···*A*
N3—H3N···N1	0.92	1.85	2.715 (8)	157
